# Revolutionizing biomolecular structure determination with artificial intelligence

**DOI:** 10.1093/nsr/nwae339

**Published:** 2024-10-10

**Authors:** Han Li, Yipin Lei, Jianyang Zeng

**Affiliations:** School of Mathematical Sciences and LPMC, Nankai University, China; Institute for Interdisciplinary Information Sciences, Tsinghua University, China; School of Engineering, Westlake University, China

Determination of biomolecular structures is crucial for understanding biological functions and designing novel therapeutics. Numerous artificial intelligence based approaches have been developed for modeling specific types of biomolecules, aiming to improve the cost and time efficiency of wet-lab experiments. Recently, the advanced deep-learning-based methods such as RoseTTAFold All-Atom (RFAA) and AlphaFold3 have emerged for generalized biomolecular structure modeling. These approaches are designed to comprehensively elucidate biomolecular interactions by accurately predicting complex structures that involve proteins and a wide range of ligands, such as small molecules, nucleic acids, ions, and modified residues, using protein sequences and ligand-specific feature representations as inputs. This perspective provides a concise overview of the evolution of biomolecular structure determination approaches, and discusses the advance and potential limitations of RFAA and AlphaFold3.

## EVOLUTION OF BIOMOLECULAR STRUCTURE DETERMINATION APPROACHES

Determination of the 3D structures of proteins and nucleic acids (i.e. DNA and RNA) is crucial for understanding their biological functions and interactions, which are essential for advancing fields such as drug discovery, molecular biology and biochemistry. These structures can be determined by using experimental techniques such as nuclear magnetic resonance spectroscopy, X-ray crystallography and single-particle cryo-electron microscopy. However, these methods are expensive and labor-intensive, and require substantial expertise, thus limiting their accessibility and application.

With the accumulation of data, as exemplified by databases such as Protein Data Bank (PDB) [1], various deep-learning-based approaches have been developed, each specializing in predicting the structures of a specific type of biomolecule. One of the most significant examples is AlphaFold2 [2], which has revolutionized protein structure modeling by providing accurate and efficient predictions of protein structures from amino acid sequences. Another notable approach, trRosettaRNA [3], has demonstrated robust and accurate predictions of RNA 3D structures from nucleobase sequences.

Beyond predicting the structures of individual biomolecules, deep-learning-based methods have also been proposed for predicting the structures of biomolecular complexes. For instance, AlphaFold-Multimer [4] is a deep-learning-based method that was designed to accurately predict the structure of protein–protein complexes, while RoseTTAFoldNA [5] specializes in predicting the structure of protein–nucleic acid complexes. In the realm of protein–small molecule complex prediction, advanced deep-learning-based docking and co-folding models such as Uni-Mol [6] and Umol [7] have increasingly surpassed traditional docking approaches [8] in both accuracy and efficiency.

**Figure 1.**
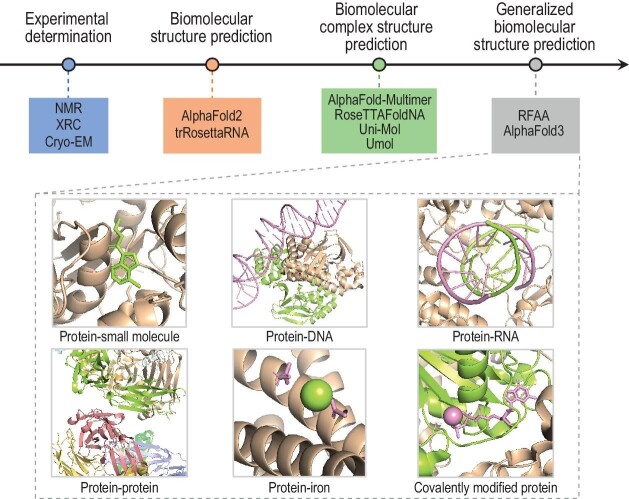
Evolution of biomolecular structure determination methods: from experimental techniques to deep-learning-based approaches for specialized biomolecular and biomolecular complex structure predictions, and generalized biomolecular structure prediction models. Key representative methods are listed below the evolution timeline. NMR, nuclear magnetic resonance spectroscopy; XRC, X-ray crystallography; cryo-EM, cryo-electron microscopy; RFAA, RoseTTAFold All-Atom.

Recently, two advanced deep-learning-based approaches—RFAA [9] and AlphaFold3 [10]—have been proposed for generalized biomolecular structure modeling (Fig. 1). Unlike earlier specialized methods, these approaches are designed to comprehensively decipher the bioactivities of various biomolecules by predicting the structures of complexes involving proteins and a wide range of ligands, including proteins, small molecules, nucleic acids, ions and modified residues.

## GENERALIZED BIOMOLECULAR STRUCTURE MODELING

Compared with specialized models, the most fundamental challenge in building a generalized model is how to accurately model different types of biomolecules within a unified framework.

RFAA is based on the three-track neural network architecture of RoseTTAFold2 [11], integrating 1D sequence information, 2D pairwise distance data and 3D coordinates of biomolecular to iteratively refine the predicted complex structures. RFAA takes input information about the molecular composition of the biomolecular complex to be modeled. For protein and nucleic acid sequences, it retains the feature representations from RoseTTAFold2 and RoseTTAFoldNA, respectively. For small molecules, covalent modifications and unnatural amino acids, RFAA represents them as atom-bond graphs, using chemical element types as the input for the 1D track, chemical bonds between atoms for the 2D track and chirality information for the 3D track. To facilitate the general biomolecular modeling, RFAA incorporates heavy atom coordinates into the 3D track in coordinate updates, enabling the representation of the full system as a disconnected gas of amino acid residues, nucleic acid bases and freely moving atoms. These input features are then progressively refined into physically plausible structures through the stacked blocks of the neural network.

AlphaFold3, which has inherited the problem formulation of AlphaFold2 and AlphaFold-Multimer, predicts the atom-level structures with confidence from the input sequence of a protein and the feature representation of its ligand, e.g. the amino acid sequence of the ligand protein, the simplified molecular input line entry system (SMILES) of the ligand small molecule and the nucleotide of the ligand DNA or RNA. To innovatively model various types of biomolecules, AlphaFold3 tokenizes their input features (individual amino acids or nucleotide residues, atom-level tokens of other biomolecules), together with pre-generated conformers of ligands, bond features, as well as the similar multiple sequence alignment (MSA) features and template features as in AlphaFold2. With the updated MSA modules and Pairformer modules, AlphaFold3 abandons the original row-wise and column-wise attention mechanisms on AlphaFold2 to save more computation and memory usage while still maintaining sufficient information about the proteins and nucleic acids. To accurately model atom-level coordinates of all-type biomolecules, AlphaFold3 exploits a non-equivariant diffusion module to directly predict atom-level structures. Given the output features from the previous modules, AlphaFold3 iteratively removes the Gaussian noise on all heavy atoms from both the atom level and the token level. Prediction of the true atomic coordinates from the fine-grained and large-scaled noisy coordinates enables the model to characterize both local and global geometric information for all-type biomolecules, which cannot be accomplished by previous approaches, such as AlphaFold2 or AlphaFold-Multimer.

By modeling a wide range of biomolecular systems with a unified model, RFAA and AlphaFold3 can acquire knowledge that generalizes to various interaction types, thereby greatly enhancing their capability in all-type biomolecular structure modeling. These models significantly outperform traditional and deep-learning-based docking tools for the structure modeling of specific biomolecular complexes, such as protein–small molecule and protein–protein complexes. Additionally, RFAA has shown its superior ability in modeling higher-order assemblies, such as structures with proteins, small molecules and nucleic acids, even without encountering such structures during training.

## CRITICAL ROLES OF TRAINING DATA

The success of deep-learning-based methods heavily depends on the quality and quantity of the training data. Compared with RFAA, AlphaFold3 employs a more comprehensive data augmentation strategy based on distillation and cropping. In addition to ground-truth structures from the PDB database, four additional training data sets for AlphaFold3 are obtained through AlphaFold distillation, including a protein monomer distillation data set derived from AlphaFold2 predictions, a disordered protein PDB distillation data set from AlphaFold-Multimer predictions, and RNA and transcription factor distillation data sets from AlphaFold3 predictions. Three main cropping strategies, including contiguous cropping, spatial cropping and interface cropping, are applied to the structures from the training data sets. While there are differences between the model architectures of AlphaFold3 and RFAA, this comprehensive data augmentation approach allows AlphaFold3 to leverage a broader range of biomolecular structures during training, significantly contributing to its enhanced performance and robustness.

Therefore, between these two models, AlphaFold3 emerges as the superior one across various tasks. Specifically, in protein–small molecule complex structure prediction, AlphaFold3 significantly outperforms all baseline methods, including RFAA, achieving a notable success rate of 76% (i.e. r.m.s.d. < 2 Å) on the PoseBusters data set [12], whereas RFAA achieves a success rate of only 42%. Although a direct comparison with RFAA in protein–nucleic complex structure prediction was not conducted, AlphaFold3 demonstrates higher accuracy than RoseTTAFoldNA, which shares a similar model architecture with RFAA.

The expansion of high-quality data is essential for further improving the performance of these models, especially for the task of RNA and protein–nucleic complex structure prediction. Currently, the available data on RNA and protein–nucleic acid complex structures are relatively limited and, as expected, the prediction performance of AlphaFold3 on these structures is less optimal compared with other tasks. Additionally, the integration of physical or experimental prior knowledge into model architectures is crucial for improving predictions in these data-scarce domains.

## ADVANCING DRUG DISCOVERY

RFAA and AlphaFold3 hold substantial promise for drug discovery, offering a more effective approach to identifying potential drug candidates. Both AlphaFold3 and RFAA outperform state-of-the-art docking tools in predicting protein–ligand interactions, indicating their potential for providing reliable information about binding sites and molecular conformations that are crucial for drug development.

The notable improvement in AlphaFold3 at predicting protein–antibody binding conformations represents a significant advancement in understanding the complexities of the human immune response and designing novel antibody-based therapeutics. Given the increasing importance of antibodies as a therapeutic modality, this enhancement underscores the broad applicability and impact of AlphaFold3 in advancing drug discovery. In addition, the capability of RFAA and AlphaFold3 to model ternary complex structures also holds great promise in aiding in the development of molecular glues.

RFAA has been released as open-source software (https://github.com/baker-laboratory/RoseTTAFold-All-Atom). AlphaFold3 has been publicly accessible via the AlphaFold Server (https://alphafoldserver.com) and is also open-source on GitHub (https://github.com/google-deepmind/alphafold3). By providing open-access tools, these two models will empower the scientific community to leverage these cutting-edge technologies without constraints. These open-source tools offer great potential to help tackle the current bottleneck of limited experimental structural data through providing a large scale of high-quality predicted structures that are useful for understanding biomolecular functions and advancing drug discovery.

## FUTURE PERSPECTIVE

The current predictions made by RFAA and AlphaFold3 are limited to static structures, overlooking the conformational diversity and changes that occur during molecular interactions, which are crucial for understanding biological processes and drug discovery. There is a need for a more advanced approach that can decode the dynamic characteristics and predict the ensemble of conformations that biomolecules adopt under various conditions.

While RFAA and AlphaFold3 have demonstrated superior capabilities in protein–ligand docking, their effectiveness in large-scale virtual screening remains unproven. This process requires the rapid and accurate assessment of thousands or millions of molecules, which is presenting significant challenges due to the diversity and complexity of the chemical space. These models must generalize across a wide range of chemotypes and binding modes to identify the most effective binders—a capability that still needs further validation for RFAA and AlphaFold3.

The computational resources and time requirements of RFAA and AlphaFold3 could potentially limit their broad applicability. While AlphaFold3 has significantly improved time efficiency by replacing the Evoformer module of AlphaFold2 with the simpler Pairformer module, a single structure prediction still takes several minutes on 16 NVIDIA A100 graphics processing units (GPUs). Although an online AlphaFold3 server is available, users are limited to just 20 requests per day, which greatly restricts its use in high-throughput scenarios. Similarly, RFAA users face challenges with the RFAA application due to limited GPU resources. In addition to accuracy and precision, deep-learning-based models must address computational resource demands and time efficiency to enhance their practical usability.

Overall, while RFAA and AlphaFold3 represent major advancements, they also underscore the need for further improvements in time efficiency, resource demands, model refinement, empirical validation and integration with other computational and experimental methods to fully unlock their potential in understanding molecular behavior and facilitating drug discovery.
